# Lithium-Ion Transport in Nanocrystalline Spinel-Type Li[In_x_Li_y_]Br_4_ as Seen by Conductivity Spectroscopy and NMR

**DOI:** 10.3389/fchem.2020.00100

**Published:** 2020-02-25

**Authors:** Maria Gombotz, Daniel Rettenwander, H. Martin R. Wilkening

**Affiliations:** ^1^Institute for Chemistry and Technology of Materials, Technical University of Graz, Graz, Austria; ^2^ALISTORE-European Research Institute, CNRS FR3104, Hub de l'Energie, Amiens, France

**Keywords:** lithium halogenides, all-solid-state batteries, ceramic electrolytes, diffusion, ionic conductivity, impedance spectroscopy, solid-state NMR

## Abstract

Currently, a variety of solid Li^+^ conductors are being discussed that could potentially serve as electrolytes in all-solid-state Li-ion batteries and batteries using metallic Li as the anode. Besides oxides, sulfides and thioposphates, and also halogenides, such as Li_3_YBr_6_, belong to the group of such promising materials. Here, we report on the mechanosynthesis of ternary, nanocrystalline (defect-rich) Li[In_*x*_Li_*y*_]Br_4_, which crystallizes with a spinel structure. We took advantage of a soft mechanochemical synthesis route that overcomes the limitations of classical solid-state routes, which usually require high temperatures to prepare the product. X-ray powder diffraction, combined with Rietveld analysis, was used to collect initial information about the crystal structure; it turned out that the lithium indium bromide prepared adopts cubic symmetry ($\text{Fd}\bar{3}\text{m}$). The overall and electronic conductivity were examined via broadband conductivity spectroscopy and electrical polarization measurements. While electric modulus spectroscopy yielded information on long-range ion transport, ^7^Li nuclear magnetic resonance (NMR) spin-lattice relaxation measurements revealed rapid, localized ionic hopping processes in the ternary bromide. Finally, we studied the influence of thermal treatment on overall conductivity, as the indium bromide might find applications in cells that are operated at high temperatures (330 K and above).

## 1. Introduction

Next-generation energy storage systems, which rely on lithium-based batteries, need to be improved in terms of energy density and safety. One possibility for developing powerful batteries is to replace the flammable F-containing liquid electrolytes usually used by ceramic electrolytes (Goodenough, 2013; Bachman et al., 2016; Janek and Zeier, 2016; Zhang et al., 2018). The thermal runaway of such batteries is expected to shift significantly toward higher temperatures.

Suitable ceramics need to show very high ionic conductivities and a sufficiently high electrochemical stability. To achieve high energy densities, the use of Li metal as anode material is necessary. Li metal has already been used as anode material (Wenzel et al., 2016) in the early stages of Li-battery research. However, stability issues, the lack of suitable (liquid) electrolytes (Bruce et al., 2008), and the formation of dendrites (Porz et al., 2017) prevented the commercialization of such systems. The discovery of highly conducting ceramics has changed this path of development. Of course, many studies have revealed that the Li|electrolyte interface suffers from contact issues (Cheng et al., 2014; Yu et al., 2017; Zhang et al., 2017). These interface issues are closely related to the formation of resistive interfacial phases (Richards et al., 2016). Controlling or preventing their formation is still a problem that needs to be overcome for the majority of ceramics currently discussed as solid electrolytes.

Presently, a range of Li-bearing oxides (Murugan et al., 2007; Buschmann et al., 2011; Thangadurai et al., 2014; Stanje et al., 2017; Uitz et al., 2017), sulfides (Kamaya et al., 2011; Wang et al., 2015; Dietrich et al., 2017), hydrides (Maekawa et al., 2009; Matsuo and Orimo, 2011; Kim et al., 2019), and thiophosphates (Deiseroth et al., 2008; Hanghofer et al., 2019) are being investigated with respect to ionic conductivity, stability and interfacial properties. Besides these classes of materials, halides (Lutz et al., 1990; Marx and Mayer, 1996; Gupta et al., 1997) are also regarded as up-and-coming materials to be employed as solid electrolytes. For instance, mixtures of Li^+^ ion conductors with lithium salts, e.g., Li_2_S mixed with LiBr, as well as compounds like LiI_1−*x*_Br_*x*_, which were already being studied in the 80s (Mercier et al., 1985; Schoch et al., 1986), show increased ionic conductivity as compared to the binary halides. The latter, particularly if we consider LiI and LiF, represent extremely poor ionic conductors. Regarding ternary Li-bearing halides with Y or In, compounds like Li_3_YBr_6_ or Li_3_YCl_6_ have entered the spotlight of research (Asano et al., 2018). Recently, the performance of a 3.5 V cell with either Li_3_YBr_6_ or Li_3_YCl_6_ as electrolyte has been investigated. First-principle analysis confirmed such compounds to have high ionic conductivity as well as relatively broad electrochemical stability. For example, a stability window of 0.59–3.15 V has been reported for Li_3_YBr_6_ (Wang et al., 2019). Because of this encouraging result, it is worth looking at this interesting class of materials and studying related compounds.

Here, we synthesized a cubic form of defect-rich Li[In_*x*_Li_*y*_]Br_4_ in a nanocrystalline form. LiInBr_4_ has been mentioned in the literature in a study carried out by Yamada et al. (2006). The authors speculated whether LiInBr_4_ should be described by (Li)_8b_[In□]_16d_Br_4_ or by (□)_8b_[InLi]_16d_Br_4_, where 8*b* and 16*d* represent the tetrahedral and octahedral voids formed by the Br anion lattice. Preliminary studies also reported on coarse-grained Li_3_InX_6_ (X = Cl, Br) (Tomita et al., 2008, 2014; Li et al., 2019). In the present case, classical solid-state synthesis routes resulted in samples with a large amount of side phases, so we decided to take advantage of a mechanochemical synthesis approach to prepare Li[In_*x*_Li_*y*_]Br_4_, which we characterized, apart from X-ray diffraction, also by high-resolution ^6^Li and ^79^Br magic angle spinning (MAS) nuclear magnetic resonance (NMR). Preliminary Rietveld analysis of the corresponding X-ray powder pattern revealed that nano-Li[In_*x*_Li_*y*_]Br_4_ most likely crystallizes with cubic symmetry; roughly speaking the stoichiometry of the sample is best described by Li[In_0.62_Li_1.38_]Br_3.92_. We used variable-temperature broadband conductivity and electrical modulus spectroscopy to measure its ionic transport properties. To understand the results from conductivity spectroscopy, we estimated site energies and diffusive barriers with the help of the bond valence energy landscape (BVEL) methodology developed by Chen et al. (2019). Here, at 293 K, the so-called direct current (dc) total ionic conductivity, σ_dc_, is given by 4.9 × 10^−6^ *Scm*^−1^. Of course, as a higher conductivity is needed for cells operated at room temperature, the ternary bromide might be a suitable electrolyte for cells that are cycled at *T*>330 *K*. Furthermore, we characterized our sample in terms of temperature stability, electronic conductivity, and Li self-diffusion. The latter was investigated by means of ^7^Li NMR line shape and spin-lattice relaxation measurements (Kuhn et al., 2011; Wilkening and Heitjans, 2012; Pecher et al., 2017; Stanje et al., 2017; Uitz et al., 2017; Dawson et al., 2018; Martin et al., 2019). Interestingly, ^7^Li NMR points to rapid localized ion-hopping processes in the nanocrystalline form of the ternary indium bromide.

## 2. Materials and Methods

### 2.1. Mechanochemical Synthesis of Nanocrystalline Li[In_*x*_Li_*y*_]Br_4_

Mechanochemical synthesis was carried out in ZrO_2_ beakers with a volume of 45 ml and using milling balls made of the same material with a diameter of 5 mm. The beakers were loaded with LiBr (99.999%, Sigma Aldrich) and InBr_3_ (99.99%, Alfa Aesar) at a ratio of 3:1. Synthesis was carried out using a high-energy planetary mill Fritsch Pulverisette 7 premium line at a milling speed of 600 rpm. The total milling time was 10 h, whereas after each milling duration of 15 min, the beakers were allowed to cool down and to rest for 15 min. Loading as well as emptying of the beakers was strictly carried out in an argon-filled glovebox (H_2_O, O_2_ < 0.5 ppm), as both the starting materials and the product are highly hygroscopic.

### 2.2. X-Ray Powder Diffraction

X-ray powder diffraction (XRPD) was carried out on a Bruker D8 Advance diffractometer [Bragg Brentano geometry, CuKα radiation (1.5406 Å)]. Between 10 and 100° 2θ data points were collected for 1 s and with a stepsize of 0.02° 2θ. The measurements, at ambient temperature, had to be performed with a sample holder designed for air-sensitive materials; here, the samples were protected from any moisture by a Kapton^Ⓡ^ foil. For reasonable background correction, we performed a reference measurement, that is, a blank run using the empty sample holder and the Kapton^Ⓡ^ foil only. X-PertHighScorePlus software (PANalytical) was used to analyze the pattern according to the method introduced by Rietveld.

### 2.3. Conductivity and Electric Modulus Spectroscopy

The overall conductivity of site-disordered Li[In_*x*_Li_*y*_]Br_4_ was investigated by broadband conductivity spectroscopy. Small amounts of the powdered sample were pressed into cylindrical pellets (5 mm in diameter) by applying a uniaxial pressure of 0.8 GPa. The pellets were equipped with ion-blocking electrodes (Pt, 100 nm in thickness) by sputtering (Leica, EM SCD050). Finally, the pellets were put in an air-tight sample holder. All preparation steps were carried out inside an Ar-filled glovebox (H_2_O, O_2_ < 0.1 ppm). Alternating current (ac) impedance data were recorded with a Novocontrol Concept 80 spectrometer in combination with an active BDS 1200 cell and a ZGS interface (Novocontrol). A QUATRO cryosystem was employed to precisely control and monitor the temperature via Pt100 thermocouples. Here, we varied the temperature *T* from −40 to 100°C. The frequency range covered for each isothermal measurement was 10^−2^–10^7^Hz.

### 2.4. Polarization Measurements to Estimate the Electronic Conductivity

The as-prepared nanocrystalline Li[In_*x*_Li_*y*_]Br_4_ was pressed into a cylindrical pellet (*d* = 8 *mm*, *h* = 1.51 *mm*) under uniaxial pressure (0.3 GPa). Pt electrodes were applied on both sides via sputtering. Due to its hygroscopic character, the pellet with this electrode sandwich configuration was mounted in an air-tight Swagelok-type cell. We prepared the pellet and the cell in a glovebox (O_2_, H_2_O < 0.1 ppm) and subsequently placed the cell in a drying oven at 60°C. The cell was then connected to a Parstat MC potentiostat (Princeton Applied Research), which is equipped with a low-current option. We applied a constant voltage *U* of 300 mV and measured the change of current *I*(*t*) over time.

### 2.5. ^6^Li and ^79^Br MAS NMR

High-resolution ^6^Li and ^79^Br magic angle spinning (MAS) NMR spectra were acquired with a 500 MHz Bruker Avance III spectrometer connected to a 11.7 T cryomagnet (Bruker Biospin, Ultrashield 500 WB plus). This magnetic field corresponds to resonance frequencies of 73.58 MHz for ^6^Li and 125.27 MHz for ^79^Br, respectively. The powder sample was stuffed into 2.5 mm ZrO_2_ rotors designed for the MAS probehead used (Bruker). The spinning speed was set to 25 kHz; spectra were recorded at a bearing gas temperature of 303 K (frame cooling: 35% target gas flow, target gas flow: 400 L h^−1^). ^6^Li MAS NMR spectra were recorded at selected temperatures viz. at 253, 273, and 303 K. We used single-pulse excitation and converted the free induction decays (FIDs) into spectra by Fourier transformation. LiBr served as reference material (0 ppm) to determine chemical shifts δ_iso_.

### 2.6. Static ^**7**^Li NMR Spectroscopy

^7^Li NMR longitudinal spin-lattice relaxation (SLR) rates (1/*T*_1_) were recorded on a Bruker Avance III spectrometer with a nominal magnetic field of 7.05 T. This field corresponds to a ^7^Li Larmor frequency of 116.59 MHz. A home-built NMR probe was used to record the static NMR signal in a temperature range from −40 to 100°C. Diffusion-induced SLR rates in the laboratory frame of reference were recorded by using a saturation recovery pulse, which includes a set of ten 90° pulses, destroying longitudinal magnetization *M*_*z*_, and delay times *t*_d_, after which the magnetization was detected with a single 90° pulse. The resulting transients *M*_z_(*t*_d_) were analyzed with stretched exponentials to determine 1/*T*_1_. The spin-lock NMR technique was used to acquire SLR_ϱ_ rates in the so-called rotating frame of reference. We used a spin-locking frequency to record ^7^Li NMR diffusion-induced rates 1/*T*_1ϱ_ at a locking field that corresponds to ω_1_/2π = 20 *kHz*. For the static ^7^Li NMR experiments, the sample was fire-sealed under vacuum in a glass (Duran^Ⓡ^) ampoule with a diameter of 5 mm and a length of ~3 cm.

## 3. Results and Discussion

### 3.1. X-Ray Diffraction and Diffusion Pathways

**Figure 1:**
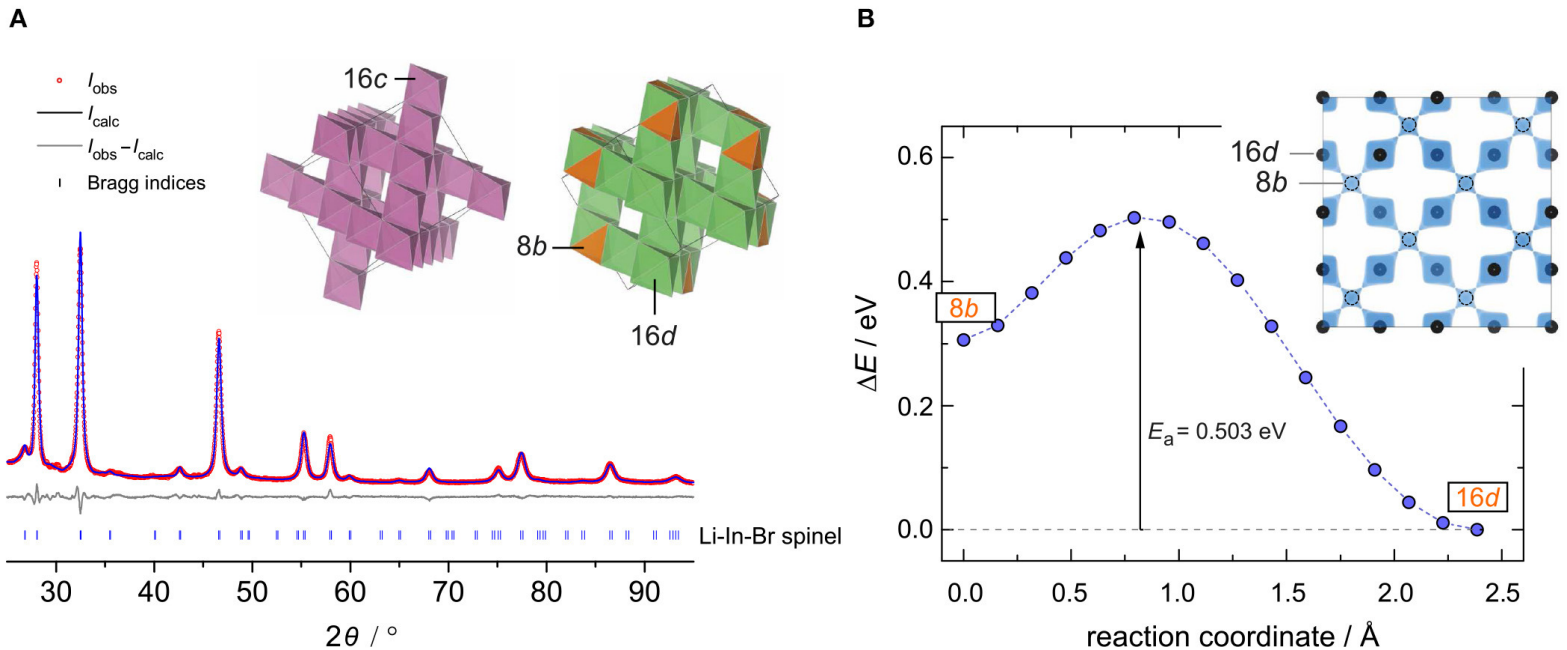
**(A)** The observed (obs) XRPD pattern as well as the calculated and difference patterns (obs-calc) of the Rietveld refinement for the intended compound Li[In_*x*_Li_*y*_]Br_4_. Vertical lines below the profile mark the positions of the possible Bragg reflections of spinel-type Li[In_*x*_Li_*y*_]Br_4_ with its site disorder. Inset: framework formed by InBr_6_ octahedra, and the Li-ion sublattice composed of tetrahedral and octahedral sites. **(B)** An energetically favored Li^+^ diffusion pathway as estimated via the BVEL method; this pathway directly links the two crystallographically positions 16*d* and 8*b*. The corresponding iso-surface is shown in the inset.

The crystal structure of nanocrystalline Li[In_*x*_Li_*y*_]Br_4_ was first characterized using XRPD. We used an airtight sample holder to record the diffraction pattern at ambient temperature; the pattern is shown in Figure 1. It perfectly agrees with that presented by Yamada et al. (2006). Our pattern reveals relatively broad reflections, which points to a sample with either low crystallinity or very small crystallites. According to the Scherrer equation (Scherrer, 1918; Heitjans et al., 2007), the mean crystallite size 〈*d*〉 is in the order of 17(5) nm if we use the reflections (448), (226), and (004) to estimate 〈*d*〉. These reflections correspond to the structure model estimated via Rietveld analysis with information derived from the bond valence energy landscape (BVEL) methodology developed by Chen et al. (2019). For our BVEL estimation, we used the structure model of Li_2_MnBr_4_. Despite the low crystallinity and the Kapton tape used to avoid degradation of the sample in air, we were able to propose a structure model (see Table 1), which summarizes the results from Rietveld refinement.

**Table 1:** X-ray diffraction data of Li[In_*x*_Li_*y*_]Br_4_ with positional disorder on the 16*d* site as determined through Rietveld refinement; space group: $Fd\bar{3}m$ (no. 227); *Z* = 8; lattice parameters: *a* = *b* = *c* = 11.026141(86) Å; *R* factors: *R*_e*xp*_ = 1.78, *R*_w*p*_ = 4.54, *R*_p_ = 3.53; goodness of fit (GoF) = 2.56.

	**Site**	***x*/*a***	***y*/*b***	***z*/*c***	***U*_iso__^a^_**	**Occ.^b^**
Li1	8*b*	1/8	5/8	1/8	4.60(31)	1.00(32)
Li2	16*d*	1/2	1/2	1/2	1.51(13)	0.69(11)
In	16*d*	1/2	1/2	1/2	1.51(13)	0.310(11)
Br	32*e*	0.25328(10)	0.25328(10)	0.25328(10)	1.316(51)	0.98(9)

a *× 100 [Å^2^]*,

b *occupation factor; 1.0 denotes full occupation of the site*.

*U_iso_ and Occ. were at first refined iteratively and finally simultaneously, leading to no change*.

*The occupation number of Li1 was set to 1, leading to an improved refinement. As Li2 and In share the 16d position, the occupation number of both together was constrained to 1*.

Indexing of all Bragg peaks is possible with the space group $Fd\bar{3}m$ (no. 227). Only a small amount of an unidentified extra phase is present. In addition, we cannot exclude that small amounts of amorphous LiBr or InBr_3_, being invisible for X-ray diffraction, are present. Because of the low scattering factor of Li, its exact assignment to crystallographic positions is difficult if not impossible for powder samples. In order to obtain an initial guess for the refinement, we calculated site energies and diffusive barriers based on the BVEL methodology (see above) (Chen et al., 2019). Although the values calculated have unclear physical meaning, they can be used to estimate cation pathways and site preferences. It turned out that the Li ions prefer to occupy the sites 16*d* (octahedral site) and 8*b* (tetrahedral site). By using this information from BVEL calculations, we were able to estimate the Li site occupancies at the Wyckoff positions 16*d* (Li2) and 8*b* (Li1) via Rietveld refinement. The site occupancies are best described by 1.38(11) per formula unit (pfu) and 1.00(32) pfu, respectively. For In, placed at 16*d*, thus sharing the same site with Li2, and Br (32*e*), we obtained 0.62(11) pfu and 3.92(9) pfu, respectively. Finally, this yields the composition Li_8b_[In_0.62_Li_1.38_]_16d_Br_3.92_, with Li-In positional disorder on 16*d*. Indium is in the oxidation state +3.

This inverse spinel-type structure displays a 3D framework consisting of edge-sharing InBr_6_ octahedra (see inset in Figure 1A). Each 16*d* site shares faces with two tetrahedral 6*b* sites, whereby each 8*b* site shares common faces with four 16*d* sites. This polyhedra connection forms a 3D diffusion pathway, as described by the calculated isosurface of the Li BVEL map (see Figure 1B). The corresponding activation energy of this pathway turned out to be ~0.5 eV. This value is in good agreement with that extracted from conductivity spectroscopy (vide infra).

### 3.2. Conductivity Measurements and Electrical Modulus

**Figure 2:**
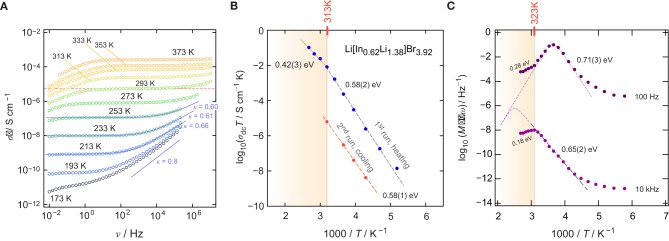
**(A)** Conductivity isotherms σ′(ν) of Li[In_*x*_Li_*y*_]Br_4_; isotherms were recorded at temperatures ranging from 173 to 373 K in steps of 20 K (1. run, heating). **(B)** Arrhenius plot to illustrate the temperature dependence of σ_d*c*_*T* for both the 1. run (heating) and the 2. run (cooling). **(C)** Real part of the complex resistivity ρ′ expressed as *M*″/ν as a function of the inverse temperature 1/*T* and recorded for a fixed frequency of either ν = 10k*Hz* or ν = 100H*z*.

To characterize long-range ionic transport in Li[In_*x*_Li_*y*_]Br_4_, we measured conductivity isotherms over a relatively broad frequency range (10^−2^–10^7^ Hz). Figure 2A shows the isotherms, that is, the real part, σ′ of the complex conductivity $\hat{\sigma }$ plotted as a function of frequency ν. The isotherms reveal three characteristic regions that are universally found (Funke et al., 2005; Dunst et al., 2014). At high temperatures and low ν, the ions pile up at the ion-blocking electrode. This charge accumulation causes the effect of electrode polarization, which is not seen at low temperatures, as ionic conductivity sharply decreases with decreasing temperature. At sufficiently high frequencies, electrode polarization passes into the so-called direct current conductivity plateau, which is characterized by σ_*dc*_, which we identify as the conductivity value that characterizes long-range ion transport. The same value could be extracted from complex plane representations of the impedance Ẑ; σ_*dc*_ refers to the intercept of the location curve with the abscissa of the corresponding Nyquist plot.

With decreasing temperature, we recognize that the dc-plateau of the isotherms shifts toward lower frequencies; simultaneously, at higher frequencies, it passes into a frequency-dependent dispersive regime. At the very low temperatures, the dispersive parts reveal a negligible temperature dependence, which indicates the beginning of the so-called nearly constant loss (NCL) regime (Dyre et al., 2009; Dunst et al., 2016). The latter is often ascribed to caged-like jump processes. While successful ion displacements govern the dc regime, the non-NCL disperse part is sensitive to unsuccessful jumps, including localized motions but also forward-backward jumps. In many cases, σ′(ν) in the dispersive regime can be approximated with Jonscher's power law σ′∝ν^κ^. κ usually takes values ranging from 0.6 to 0.9 if ionic transport occurs in three dimensions. 1D and 2D ionic transport might result in lower values (Sidebottom, 2009). Here, we found κ ≈ 0.6 (253 K), pointing to 3D ionic transport (see Figure 2A), as expected from the BVEL estimation (*vide supra*).

σ_dc_ values were directly read off from the distinct dc-plateaus of Figure 2A. σ_dc_*T*, plotted vs. 1000/*T*, is shown in Figure 2B. The linear behavior seen for the 1. run, which corresponds to the heating run, points to Arrhenius behavior according to σ_dc_*T* = σ_0_exp(*E*_a_/(*k*_B_*T*)). Here, *k*_B_ denotes Boltzmann's constant and σ_0_ represents the Arrhenius pre-factor. The activation energy *E*_a_ turned out to be 0.58(2) eV. Above 313 K, a kink is seen and, at higher *T* than 313 K, the activation energy reduces to 0.42(3) eV. *E*_a_≈0.58 *eV* (193–313 K) agrees nicely with the value estimated from BVEL (0.5 eV, see above).

The origin of the change of *E*_a_ at 313 K remains so far unknown; it was also observed by Yamada et al. (2006). If no phase transition takes place in Li[In_*x*_Li_*y*_]Br_4_, one might assume that an order-disorder transition could cause this kink in the Arrhenius line. Such a transition has also been observed for Li_2_MnBr_4_ and Li_2_MgBr_4_ but at higher temperatures (Schmidt and Lutz, 1984). In addition, above 313 K, grain growth may already start, which is accompanied by the healing of, for example, point defects. It is well-known than the ionic self-diffusivity in poorly conducting materials can be drastically increased by the introduction of defects, either through mechanical treatment or through radiation (Stathopoulos and Pells, 1983; Ishimaru et al., 2003). Hence, at temperatures above 313 K, the material continuously changes and transforms into a more ordered structure. As expected, the conductivity values recorded during the 2. run, that is, the cooling run (313 K → 233 K), turn out to be lower by ~3 orders of magnitude as compared to that governing the 1. run.

**Figure 3:**
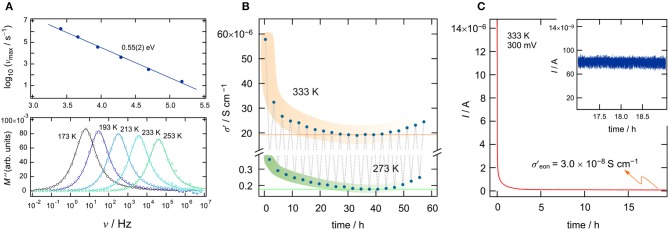
**(A)** The upper graph shows the Arrhenius behavior of the characteristic frequencies ν_*max*_ at which the *M*″ peaks appear. Frequency dependence of the imaginary part of the complex part of the modulus as a function of frequency (ν). **(B)** Longtime measurement of σ_d*c*_ of Li[In_*x*_Li_*y*_]Br_4_ over a time period of 58 h; we cycled the temperature in the sample chamber between 273 and 333 K. **(C)** Potentiostatic polarization curve (*U* = 300 m*V*) of Li[In_*x*_Li_*y*_]Br_4_ sandwiched between two Pt electrodes. The *I*(*t*) curve was recorded at *T* = 333 K. After *t* = 18 h, a final current of *I*_final_ = 8 × 10^−8^ A has been reached (see also inset), which leads to an electrical conductivity of σ_e*on*_ = 3 × 10^−8^Scm^−1^.

To investigate ionic transport further by means of electric modulus formalism (Hanghofer et al., 2019), we recorded the quantity *M*″/ν (see also Figure 2C) as a function of temperature and for constant frequency (ν = 100 *Hz* and ν = 10 *kHz*, 173 K ≤ *T* ≤ 373*K*). It turned out that, beginning from very low *T*, the quantity *M*″/ν obeys a weaker-than-activated temperature behavior, in agreement with NCL-type electrical relaxation processes. Above 250 K, we recognize a strong increase with the inverse temperature. From the linear behavior observed, we extracted activation energies of 0.65(2) eV (10 kHz) and 0.71(3) eV (100 Hz). The increase seen if we compare these values to that found by σ_dc_ [0.58(2) eV] indicates that the number of charge carries seems to depend on temperature. Again, above 313 K, and independent of frequency ν, we observe a drastic change in *M*″/ν(1/*T*). The corresponding apparent activation energy of ~0.18 eV (and 0.28 eV, see Figure 2) is much smaller than that seen in conductivity spectroscopy. We attribute this kink and the associated decrease in *E*_a_ to changes in morphology and the defect chemistry of the bromide. Finally, we looked directly at the temperature dependence of the electric modulus *M*″(ν); the corresponding modulus peaks are shown in the lower part of Figure 3A. The Arrhenius behavior of the characteristic frequencies ν_max_ at which the *M*″ peaks appear is displayed in Figure 3A. The linear fit points to an activation energy of 0.55 eV, which is smaller than that seen by σ_dc_. The lower activation energy again points to a *T*-dependent charge carrier concentration *N*^−1^ that increases with temperature. As σ_dc_ is directly proportional to the charge carrier mobility *mu*_*q*_, the charge of the moving species *q* and *N*^−1^, ν_max_, being a characteristic electrical relaxation frequency, increases slightly less with *T* than σ_dc_.

At 293 K, we obtain an ionic conductivity of σ_dc_ = 4.9 × 10^−6^*Scm*^−1^; this value is far below values needed for ceramic electrolytes in all-solid-state batteries. Thus, the current material is expected to be used in systems operated at higher *T*. For this reason, we studied the temperature stability of the ionic conductivity of the defect spinel over a time period of 58 h between 273 and 333 K. Moreover, as a sharp decrease in ionic conductivity was observed during the cooling run, we were interested in finding out which final conductivity value will be reached after annealing the sample for many hours at 333 K, which is just slightly above the kink seen in Figure 2. Hence, we kept the sample at 333 K for 1 h and then measured σ′(ν). The time needed to record the isotherm was 23 min. After that, we decreased the temperature in the sample chamber to 273 K, waited 1 h, and measured another isotherm σ′(ν); this cycle was repeated several times (see Figure 3B). For both the measurements at 333 K and at 273 K, we see the same trend. The conductivity decreases and reaches an almost constant value of σ_dc_ = 2 × 10^−5^*Scm*^−1^ at 333 K. The corresponding conductivity at 273 K is two orders of magnitude lower as compared to that measured at 333 K. The reason for the slight increase in conductivity with increasing cycle number is still unknown.

Apart from overall conductivity and thermal stability, the ability of a material to conduct electrons is of significant importance when it comes to its application (Han et al., 2019). Ideally, the electronic conductivity should be orders of magnitude lower than the ionic contribution to the overall conductivity. Only electronically insulting ceramics will prevent soft shorts or the formation of detrimental Li dendrites. Here, we used a symmetric Pt ∣ Li[In_*x*_Li_*y*_]Br_4_ ∣ Pt cell to monitor the evolution of current *I*(*t*) at a constant voltage *U* = 300 *mV*. The final current of such a polarization measurement (Breuer et al., 2018a; Gombotz et al., 2019) will give the upper limit of the electronic conductivity σ_eon_. The curve shown in Figure 3C refers to 333 K and reaches a steady state of *I* after 18 h. The final current (*I*_final_ = *I*(18h)) can be used to estimate σ_eon_ according to σ_eon_ = $I_{\text{final}}/U\cdot d/\left(r^{2}\pi \right)$, *d* denotes the thickness of the pellet and *r* refers to its diameter. For σ_eon_ we obtain σ_eon_ = 3 × 10^−8^*Scm*^−1^ at 333 K, which is a factor of 10^3^ smaller than the overall conductivity at this temperature ($\sigma_{\text{dc}}\approx 6\times 10^{-5}Scm^{-1}$). For battery applications, even smaller values for σ_eon_ would be desirable ($\sigma_{\text{dc}}/\sigma_{\text{eon}}>10^{6}$) and, thus, great care has to be taken when In-containing ceramics play a role as solid electrolytes. As an example, for oxide electrolytes, values of σ_eon_ in the order of 10^−8^*Scm*^−1^ have been reported to trigger Li dendrite formation in all-solid-state batteries (Han et al., 2019). Li dendrites constitute a major safety problem, as they initiate short circuits and, thus, thermal runaways.

### 3.3. ^**7**^Li NMR Spin-Lattice Relaxation and Line Shapes

**Figure 4:**
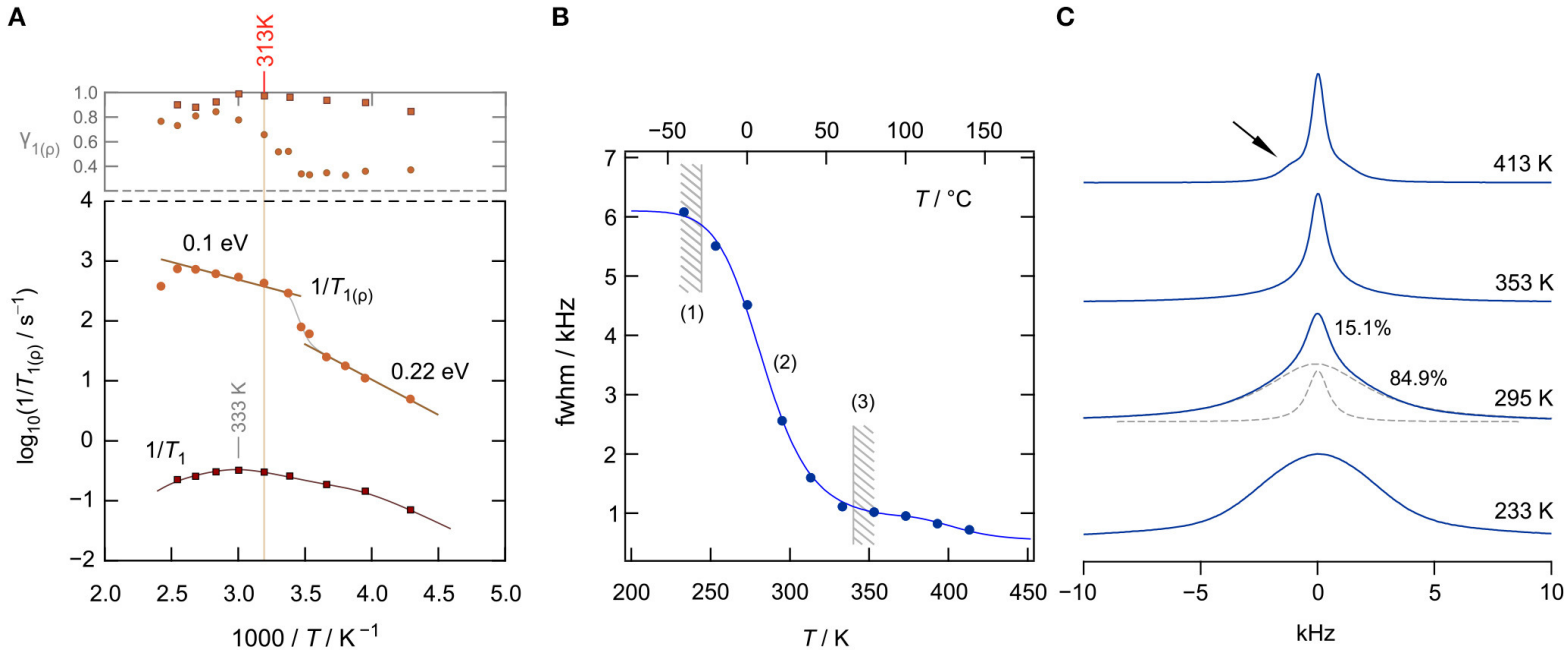
**(A)** ^7^Li NMR spin lattice relaxation (SLR) rates 1/*T*_1(ϱ)_ (116.59 MHz) of nanocrystalline, cubic-Li[In_*x*_Li_*y*_]Br_4_ as a function of inverse temperature measured (233–393 K). The upper graph shows the γ_1(ϱ)_ values used to approximate the magnetization transients. **(B)** ^7^Li NMR line width (fwhm = full width at half maximum, total fwhm from net spectrum) as a function of temperature; regime (1) denotes the rigid lattice, (2) refers to the motional narrowing regime, and (3) represents the regime of extreme narrowing. The solid line (up to 353 K) represents a fit according to the model Abragam, yielding an activation energy of ~0.25 eV. **(C)** Change of the shape of the ^7^Li NMR spectra with temperature (233, 295, 353, and 413 K). The spectrum at 295 K has been deconvoluted with a sum of a Gaussian and a Lorentzian function (see dashed lines). Motional narrowing is almost completed at *T* = 353 K.

To study solely ion dynamics in Li[In_*x*_Li_*y*_]Br_4_, we took advantage of ^7^Li NMR SLR measurements. In general, NMR spectroscopy is a contactless method and does not require any post-preparation steps, such as the application of conducting electrodes. As it is mainly sensitive to bulk ion dynamics, any interfering effects of ion-blocking grain-boundary regions are absent. Of course, the latter holds only for single crystals or polycrystalline materials with crystallites that show diameters in the μ*m* range. For nanocrystalline samples, the ions residing in the large volume fraction of interfacial regions can be, simultaneously with those in the bulk regions, visualized by NMR (Wilkening et al., 2003; Breuer et al., 2018b). Here, we recorded diffusion-induced ^7^Li SLR rates, in both the laboratory and rotating frame of reference, as a function of the inverse temperature (233–393 K) (Heitjans et al., 2005). The magnetization transients were analyzed in terms of stretched exponential functions (Stanje et al., 2017). The stretching exponents γ_1(ϱ)_ are given in the upper graph of Figure 4A; the corresponding rates are shown in the Arrhenius diagram below.

Interestingly, the SLR NMR rates 1/*T*_1_ of nano-Li[In_*x*_Li_*y*_]Br_4_ reveal only a slight dependence on reciprocal temperature. We recognize shallow maxima at ca. 333 K and ca. 260 K. Usually, if purely induced by diffusion, the rate 1/*T*_1_(1/*T*) passes through a rate peak whose flanks contain the activation energy. For 3D uncorrelated motion, a symmetric peak is expected (Bloembergen et al., 1948); correlated motion, which originates from structural disorder and/or Coulomb interactions, often produces an asymmetric peak with a lower slope in the limit ω_0_τ ≫ 1, that is, on the low-*T* side of the peak. The same asymmetry is obtained for ionic diffusion in an irregularly shaped potential landscape, with short-range motions being different from long-range ionic transport. In the limit ω_0_τ ≫ 1, the flank contains a mean activation energy that characterizes short-range diffusion, i.e., localized site-to-site hopping processes. In the limit ω_0_τ≪1, that is, the high-*T* side of the peak, the slope of the flank contains an average activation energy that might be identified with that sensed by σ_dc_. Here, the shallow peaks are characterized by very small values of *E*_*a*_≈0.1 *eV*. Thus, they seem to sample localized ionic motions, which could also originate from Li spins in the interfacial regions (Wilkening et al., 2003; Breuer et al., 2018b) of nanocrystalline Li[In_*x*_Li_*y*_]Br_4_. Note that at above 313 K, the Arrhenius line of σ_dc_*T* reveals a kink. Thus, care has to be taken if we want to interpret the rates 1/*T*_1_ recorded at *T*>313 *K*; they might be affected by irreversible change in the nanostructured sample, which produces only an apparent rate peak.

To study ^7^Li NMR SLR behavior at temperatures well below 313 K, we recorded spin-lock NMR rates 1/*T*_ϱ_ in the rotating frame of reference. These rates sense, *per se*, slower ion dynamics as, formally, the Larmor frequency in the MHz range is replaced by a value in the kHz range. Here, we used a locking *B*_1_ field that corresponds to a spin-lock frequency of 20 kHz. The rates obtained are also included in Figure 4A. At low *T*, they follow an activation energy of 0.22 eV, which we attribute to the elementary hopping barrier in site-disordered Li[In_*x*_Li_*y*_]Br_4_, also including jump processes in the interfacial regions of the sample. If considering the crystal structure of spinel-type Li[In_*x*_Li_*y*_]Br_4_, 1/*T*_ϱ_ seems to sense (local) hopping between the sites 16*d* and 8*b*. At higher temperatures, the rate shows a sharp increase, passing into a region that is activated with an activation energy of only 0.1 eV. Again, irreversible changes in the heat-sensitive sample seem to be responsible for the anomalous behavior appearing at elevated *T*.

Alternatively, Li ion dynamics in the ternary indium bromide were studied by recording ^7^Li NMR spectra; selected spectra are displayed in Figure 4C). At low *T*, that is, the so-called rigid-lattice regime, Li^+^ motions are too slow to effectively average dipole-dipole interactions. Thus, the line can be well-described with a broad Gaussian. With increasing *T*, we clearly see the onset of motional line narrowing. This narrowing process proceeds in a heterogeneous way, i.e., a narrow line appears on top of the broad one. At *T* = 295 *K*, the area under the narrow line amounts to about 15%, showing that only a fraction of the available Li spins participate in rapid ion exchange. As for other nanocrystalline ceramics, one might attribute this fraction to those spins residing in the structurally disordered interfacial regions (Wilkening et al., 2003; Breuer et al., 2018b) of nanocrystalline Li[In_*x*_Li_*y*_]Br_4_. At elevated *T*, the whole line is affected by motional narrowing. The regime of extreme narrowing is reached at 350 K.

At even higher temperatures, intensities next to the central line appear (see arrow in Figure 4C), which we attribute to quadrupole satellites. Likely, grain growth and healing of defects ensure the formation of an ordered phase. In nanocrystalline or amorphous ceramics, satellite intensities are usually smeared out and only appear as a broad foot. Distinct singularities, referring to spin-transitions between the Zeeman levels characterized by *m*_*I*_ = ±1/2 and *m* = ±3/2, are seen in samples with a high degree of crystallinity. Here, σ_dc_ of the annealed sample is, however, much lower than σ_dc_ of the as-prepared, mechanosynthesized sample (see Figure 2B). The full narrowing curve of the ^7^Li NMR central transition is shown in Figure 4B. The solid line shows a fit with the model introduced by Abragam (1961) to parameterize the curve; the activation energy turned out to be 0.25 eV, in agreement with the value seen by spin-lock NMR. Details on analyzing NMR line widths with the Abragam formula are given elsewhere (Wilkening et al., 2002). Interestingly, at higher *T*, a second step in the motional narrowing curve is seen, which is in line with the deviations seen by σ_dc_ measurements and by the analysis of *M*″/ν data (see above).

**Figure 5:**
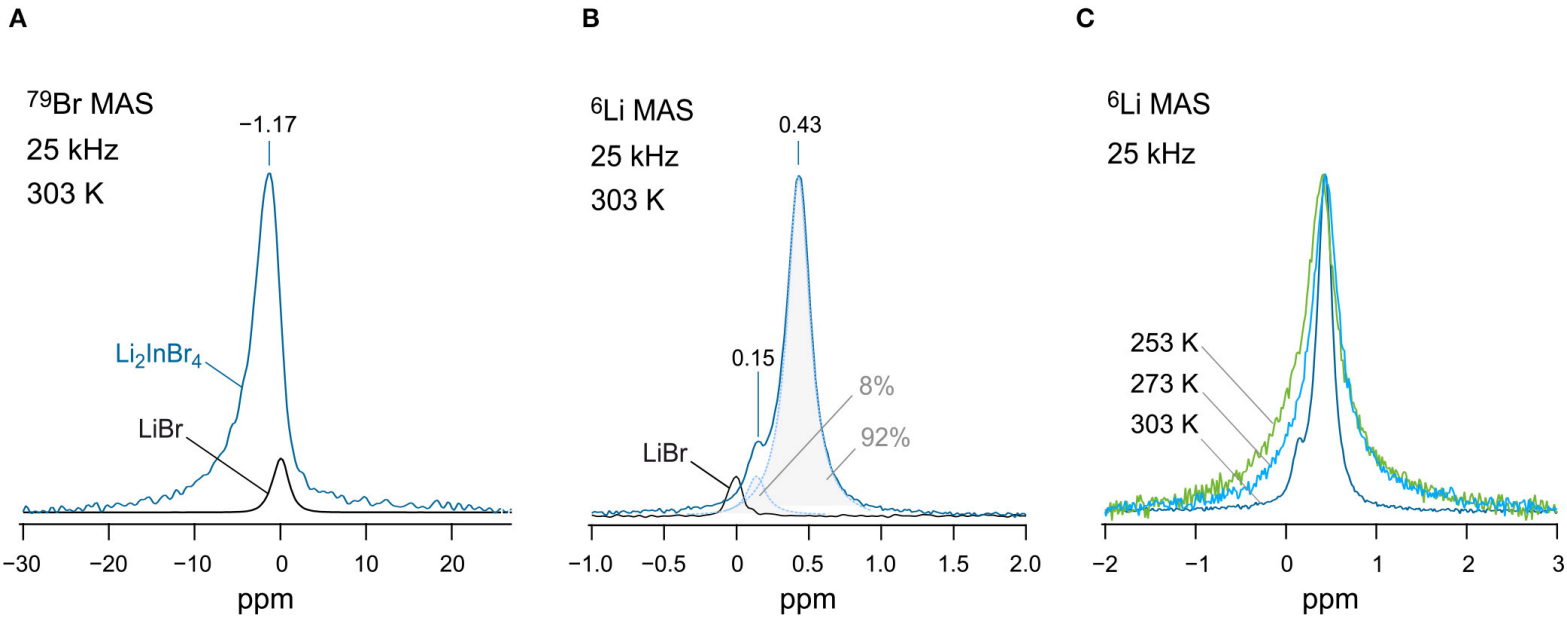
**(A)** ^79^Br MAS NMR spectra (125.27 MHz) of nano-Li[In_*x*_Li_*y*_]Br_4_ and pure LiBr for comparison. Spectra were recorded at 303 K. LiBr served as a primary reference for both the ^79^Br MAS NMR and ^6^Li MAS NMR spectra, see **(B)**. **(B)** ^6^Li MAS NMR spectra (73.58 MHz) of the nanocrystalline sample; the signal of pure LiBr is shown for comparison. The overall signal of Li[In_*x*_Li_*y*_]Br_4_ is composed of two lines appearing at 0.43 and 0.15 ppm. The total NMR signal was deconvoluted with the help of two Lorentzian functions to estimate the area under the signal with low intensity (8%). **(C)** Variable-temperature ^6^Li MAS NMR spectra (73.58 MHz) recorded at 253, 273, and 303 K. Dipolar interactions cause line broadening at low temperatures.

Finally, we checked whether it is possible to distinguish any magnetically inequivalent Li sites by ^6^Li high-resolution (MAS) NMR. For this purpose, we recorded variable-temperature ^6^Li NMR spectra (Figures 5B,C). Note that the quadrupole moment of the ^6^Li nucleus is a factor of 50 smaller than that of the ^7^Li one. Thus, any interfering second-order quadrupole interactions, which are not averaged under ordinary MAS conditions, are almost eliminated, leading to a higher resolution of ^6^Li NMR spectra. At 253 and 273 K, we detected only a single ^6^Li line at δ_iso_ = 0.43 *ppm*. We used LiBr (0 ppm) as a primary reference of the spectra. At 303 K, however, increasing dipole-dipole averaging originating from motional narrowing, which adds up to the elimination of dipolar broadening by MAS, reveals a second line at δ_iso_ = 0.15 *ppm*. At first glance, we would simply assign this additional line to LiBr. The area under the line with low intensity amounts to ~8% if we convolute the total signal with a sum of two Lorentzian functions. Interestingly, the line at 0.15 ppm does not match the isotropic shift of LiBr exactly. Furthermore, ^79^Br MAS NMR gives no strong evidence for LiBr being indeed detectable by NMR (see Figure 5A). The signal with low intensity might be attributed to the unidentified phase seen in XRD. Possibly, the presence of In-doped LiBr could serve as an explanation of the signal.

## 4. Conclusion

We successfully synthesized nanocrystalline Li[In_*x*_Li_*y*_]Br_4_ directly via a one-pot mechanochemical route. The nanostructured sample was characterized by X-ray powder diffraction and ^6^Li, ^79^Br MAS NMR. Li[In_*x*_Li_*y*_]Br_4_ needs to be described by a spinel structure with positional disorder on the 16*d* site. Via the bond valence energy landscape methodology, we estimated site energies and a hopping barrier between the Li sites 16*d* and 8*b*. Broadband conductivity helped us to measure long-range ion transport that is characterized by an activation energy of 0.61 eV. Electric modulus data pointed to barriers as high as 0.71 eV if analyzed at 100 Hz. Most likely because of the non-stoichiometry of the sample, a relatively high electronic conductivity in the order of 10^−8^ *Scm*^−1^ has been found, which is a factor of 10^3^ lower than the room-temperature ionic conductivity. Local barriers were probed by diffusion-induced ^7^Li NMR SLR measurements. In particular, spin-lock NMR yielded an activation energy of 0.22 eV, which either characterizes local (forward-backward) Li jumps between 16*d* and 8*b* or motional events of the Li ions in the interfacial regions. As σ_dc_ is rather low at room temperature, Li[In_*x*_Li_*y*_]Br_4_ seems to be a candidate for high-temperature battery cells.

## Data Availability Statement

The raw data supporting the conclusions of this article will be made available by the authors, without undue reservation, to any qualified researcher.

## Author Contributions

MG: synthesis of the material via the mechanochemical synthesis route, characterization, evaluation and interpretation of the data, and writing. DR: rietveld refinement, Li pathway calculations, and data interpretation. HW: supervision, conceptualization, data interpretation, and writing. All authors contributed to the manuscript revision, read, and approved the submitted version.

### Conflict of Interest

The authors declare that the research was conducted in the absence of any commercial or financial relationships that could be construed as a potential conflict of interest.
